# Hepatitis B Virus (HBV), Hepatitis C Virus (HCV) and Human Immunodeficiency Virus (HIV) infections among undocumented migrants and uninsured legal residents in the Netherlands: A cross-sectional study, 2018–2019

**DOI:** 10.1371/journal.pone.0258932

**Published:** 2021-10-29

**Authors:** Sarineke Klok, Eline van Dulm, Anders Boyd, Ellen Generaal, Sally Eskander, Ivo Kim Joore, Brigitte van Cleef, Evelien Siedenburg, Sylvia Bruisten, Yvonne van Duijnhoven, Gerdien Tramper-Stranders, Maria Prins

**Affiliations:** 1 NGO health care clinic Kruispost, Amsterdam, The Netherlands; 2 Department of Infectious Diseases, Public Health Service Amsterdam (GGD), Amsterdam, The Netherlands; 3 HIV Monitoring Foundation, Amsterdam, The Netherlands; 4 Department of Infectious Diseases, Public Health Service Flevoland, Lelystad, The Netherlands; 5 Department of Pediatrics, Franciscus Gasthuis & Vlietland, Rotterdam, The Netherlands; 6 Division of Infectious Diseases, Department of Internal Medicine, Amsterdam UMC, Univ. of Amsterdam, Amsterdam, The Netherlands; 7 Amsterdam Institute for Infection and Immunity (AI&II), Amsterdam UMC, Univ. of Amsterdam, Amsterdam, the Netherlands; Centers for Disease Control and Prevention, UNITED STATES

## Abstract

**Background:**

Migrants are not routinely screened for hepatitis B virus (HBV), hepatitis C virus (HCV) and human immunodeficiency virus (HIV) in the Netherlands. We estimated the prevalence and determined factors associated with HBV, HCV and/or HIV infections among undocumented migrants and uninsured legal residents.

**Methods:**

In this cross-sectional study, undocumented migrants and uninsured legal residents were recruited at a non governmental organization (NGO), healthcare facility in the Netherlands and were invited to be tested for hepatitis B surface antigen (HBsAg), anti-hepatitis B core antibodies (anti-HBcAb), HCV-RNA, and anti-HIV antibodies or HIV antigen at a local laboratory.

**Results:**

Of the 1376 patients invited, 784 (57%) participated. Participants originated from Africa (35%), Asia (30%) and North/South America (30%). 451/784 (58%) participants went to the laboratory for testing. Of participants 30% were HBV exposed (anti-HBcAb-positive), with 27% (n = 119/438, 95% CI 23.1% to 31.6%) having resolved HBV infection (HBsAg-negative) and 2.5% (n = 11/438, 95%CI 1.3% to 4.5%, 64% new infection) having chronic HBV infection (HBsAg-positive). Compared to HBV non-exposed, HBV exposed individuals were older (*p* = 0.034) and more often originated from Africa (*p*<0.001). Prevalence of chronic HCV infection (HCV-RNA-positive) was 0.7% (n = 3/435, 95%CI 0.1% to 2.0%, all new infections) and HIV infection 1.1% (n = 5/439, 95%CI 0.04% to 2.6%, 40% new infection).

**Conclusion:**

Prevalence of chronic HBV, chronic HCV and HIV infections in our study population is higher compared to the Dutch population, thus emphasizing the importance of case finding for these infections through primary care and public health in this specific group of migrants. Screening uptake could be improved by on-site testing.

## Introduction

In the Netherlands, the estimated prevalence of chronic hepatitis B virus (HBV) infection is 0.34% and of chronic hepatitis C virus (HCV) infection 0.16% in 2016. Prevalence of human immunodeficiency virus (HIV) infection was estimated at 0.14% in 2017. Migrants account for 81% of prevalent chronic HBV infections, 60% of prevalent chronic HCV infection [1–3] and 50% of prevalent HIV infections [mostly among men who have sex with men (MSM)] [2,4]. Nevertheless, the prevalence of these infections in migrants depend on a variety of factors, such as the endemicity in their country of origin and susceptibility of acquiring infection while travelling to and staying in Europe [5–7].

Asylum seekers and migrants are not routinely screened for HBV, HCV and HIV in the Netherlands [8]. Guidelines for general practitioners (GP) recommend *case finding* for infectious diseases during GP consultations, depending on the endemicity of the migrant’s country of origin [9]. However, provider- and patient-related barriers, such as competing priorities and fear of requesting testing, make these recommendations difficult to routinely implement [10]. Furthermore, HBV, HCV and HIV follow a prolonged, asymptomatic phase during the natural course of their infections, which might not lead to suspected infection during routine consultations and thereby could delay diagnosis [11]. Earlier diagnosis could help migrants obtain treatment to control HIV or HBV replication [12] or cure HCV with highly effective, direct-acting antivirals [13]. In recent years, several screening programs have targeted migrant and other key groups to find undiagnosed cases of HBV, HCV and HIV infections [14]. Both integrated (i.e. case finding in the context of other access points to healthcare) and non-integrated screening programs have been shown to be successful, depending on their intended goal [14]. For instance, a study from Bil et al. examined whether integrated screening for HBV, HCV and HIV infections at the tuberculosis (TB) clinics of the Public Health Service of Amsterdam and Gelderland was feasible and effective and although its implementation was successful, no newly diagnosed HIV or chronic HCV infections were found in Amsterdam, whereas all infections were newly diagnosed in Gelderland [15].

Undocumented migrants (including rejected asylum seekers, migrants with expired visas and ‘directly undocumented migrants’, i.e. those who bypassed asylum procedures [16,17]) and uninsured legal residents are thought to represent a considerable fraction of migrants residing in the Netherlands, yet the exact proportion is unknown. In addition, little is known about the infectious disease status of undocumented migrants and uninsured legal residents in the Netherlands A study conducted in Italy found a higher prevalence of HBV, HCV and HIV infections among undocumented migrants compared to the local population [18]. Another study in Denmark observed a higher prevalence of these infections in undocumented compared to documented migrants [19]. These differences in prevalence underline the importance of studying undocumented migrant populations.

This study aimed to assess the prevalence of HBV (chronic or resolved), HCV (chronic or resolved) and HIV infections among undocumented migrants and uninsured legal residents in Amsterdam, the Netherlands. We also intended to study the determinants of infection in this population. Additionally, we determined screening uptake, outcome of referral to specialized care (for those with infection) and HBV vaccination uptake (for non-exposed individuals).

## Methods

### Study population, design and eligibility criteria

This cross-sectional study was performed at Kruispost, a low-threshold NGO primary care facility for undocumented migrants, homeless and/or uninsured individuals. Data were used from a convenience sample of 1000 patients visiting Kruispost for a general practitioner (GP) consultation between October 2018 and October 2019. Visitors aged 18 years or older who were able to understand study information (provided in Dutch, English, French, Spanish, Arabic and Portuguese) were eligible for participation. Prior to June 20, 2019, we included patients who were born in a country outside of the European Union (EU) or European Economic Area (EEA), or those who were in possession of a citizen service number (CSN) when born in a country within the EU/EEA. After June 20, 2019, patients from a country within the EU/EEA without a CSN were also asked to participate due to a policy change made by the Dutch government that allowed care and treatment reimbursement for this group.

### Ethical statement and screening procedure

This study was carried out according to the ethical guidelines of the 1975 Declaration of Helsinki. The medical ethics committee of the Amsterdam University Medical Center (location AMC) decided that the study did not pertain to the Medical Research Involving Human Subjects Act (WMO) and therefore did not require institutional review board approval (W18_164#18.202). After general practitioner (GP) consultation, eligible patients were asked to participate in the study and were provided with study information. Many undocumented migrants are uncomfortable with providing their written name or signature on paper and therefore we requested that all participants provide verbal informed consent to participate. When verbal informed consent was given, it was recorded in our electronic data capture system. All participants were able to withdraw consent at any time during the study without clarification. For those who did not participate, we asked them to fill out a short questionnaire on basic characteristics and reason for non-participation. Data were pseudonymized for further analysis. Participants verbally completed questionnaire 1 (S1 File) together with assistance of the researcher. In questionnaire 1, lifetime risk factors for HBV or HCV infection (admission or treatment in a foreign hospital, surgery abroad, blood transfusion, paying or being paid for sex, sexual contact with men and/or women and injecting drug use) were asked. Afterwards, participants independently filled out questionnaire 2 (S2 File) in which questions were asked about sociodemographic variables (age, sex, country of birth, educational level) and migration history (year of leaving country of origin, year of arrival in the Netherlands, way of entering the Netherlands, housing situation, the number of housemates currently living with).

We conducted the study in accordance with the current screening recommendations from the Dutch government. HIV screening is universal; however, screening HBV and HCV is conducted only if individuals present with risk-factors for viral hepatitis infection (i.e. case finding) [9]. Accordingly, all participants were offered testing for HIV. The researcher determined whether risk factors for HBV and HCV infection were present based on the respondent’s answers to questionnaire 1. If so, the participant was offered testing for both HBV and HCV infections. Participants received one (for HIV testing) or two laboratory forms (for HIV and HBV/HCV testing). With these forms, participants could get tested free of charge at one of 39 blood sampling locations in Amsterdam (*Atalmedial*). Participants could refuse testing for any of the three infections. All participants were offered an incentive for their participation (a ticket for public transport, socks, disinfectant hand soap and shampoo).

### Laboratory analysis

Blood serum samples were first tested for hepatitis B surface antigen (HBsAg), anti-Hepatitis B core (Anti-HBc) antibodies, anti-HCV antibodies, and HIV antigen or antibodies (LIAISON XL MUREX, DiaSorin, Italy) at the Public Health Laboratory of GGD Amsterdam. Samples testing positive for HBsAg or anti-HBc antibodies were further tested for hepatitis B ‘e’ antigen (HBeAg), anti-Hepatitis B ‘e’ antibodies (anti-HBeAb) and anti-HBs antibodies (LIAISON XL, DiaSorin, Italy). Participants with samples testing positive for anti-HCV antibodies were asked for a second blood draw, from which anti-HCV antibody positive status was confirmed and HCV RNA was tested at the clinical virological laboratory of the Amsterdam UMC (HCV Quantitative test, version 2.0, detection limit 10–50 copies/ml, Cobas AmpliPrep/Cobas TaqMan, Roche, Switzerland). Samples testing positive for HIV antigen or anti-HIV antibodies were confirmed by Western blot (INNO-LIA HIV I/II Score, Innogenetics, Belgium) and HIV-1 p24 antigen test (Vidas HIV P24, bioMérieux).

Participants with anti-HBc antibody-positive serology were defined as having been exposed to HBV (i.e. HBV exposed). Based on HBsAg results, these participants were further classified as having resolved infection (HBsAg-negative) or chronic infection (HBsAg-positive). Participants with anti-HCV antibody positive serology were defined as having been exposed to HCV (i.e. HCV exposed). Based on HCV RNA results, these participants were further classified as having resolved infection (undetectable HCV RNA) or chronic infection (detectable HCV RNA). Participants with confirmed HIV antigen- or antibody-positive tests were considered HIV-positive.

### Follow-up care and treatment

Participants who did not have chronic HBV infection, chronic HCV infection or HIV infection were notified of their results during a telephone call. Participants who did have HBV, HCV and/or HIV infection were verbally informed of their test results by the GP. If necessary, participants were referred to the nearest hospital in Amsterdam for care and treatment, per Dutch GP Guidelines. To determine liver fibrosis levels, transient elastography was measured by a trained technician using FibroScan® and values were converted into METAVIR equivalents. Individuals who were HBsAg-negative and anti-HBc antibody-negative (i.e. non-exposed) were proposed to receive HBV vaccination at the Public Health Service of Amsterdam provided that they were eligible [20] (i.e. MSM and/or participants ever having been paid for sex). These participants received information about free HBV vaccination by telephone from a researcher at the Public Health Service of Amsterdam. In addition, they could discuss HBV vaccination during their consultation with the GP at Kruispost. Information about follow-up and treatment was extracted from the electronic patient files of the GP.

### Statistical analyses

Sociodemographic and risk factors for HBV/HCV/HIV were summarized by testing uptake. Comparisons between groups were made using Pearson χ^2^ or Fisher Exact test for categorical data and by Mann-Whitney U test for continuous data. Prevalence of HBV, HCV and HIV and its corresponding Clopper-Pearson 95% confidence interval (CI) were calculated. We compared characteristics across groups of HBV (no infection, resolved infection, chronic infection), HCV (no infection, resolved infection, chronic infection) and HIV (HIV-negative, HIV-positive) infection. Due to the limited number of infections, we could only perform risk-factor analysis for patients who were HBV exposed versus HBV non-exposed. Odds ratios (OR) comparing odds between HBV exposed and non-exposed across levels of determinants, along with their 95%CI, were calculated using univariable logistic regression. All variables with an associated *p*-value <0.2 in univariable analyses were included in a full multivariable model. A backwards-stepwise selection procedure was performed, whereby variables with the highest *p*-value were consecutively removed until only those with a *p*<0.05 remained. Significance level was set at *p*<0.05. All analyses were conducted with Stata 15.1 (StataCorp., College Station, Texas, USA).

## Results

### Characteristics of participants

**Fig 1:**
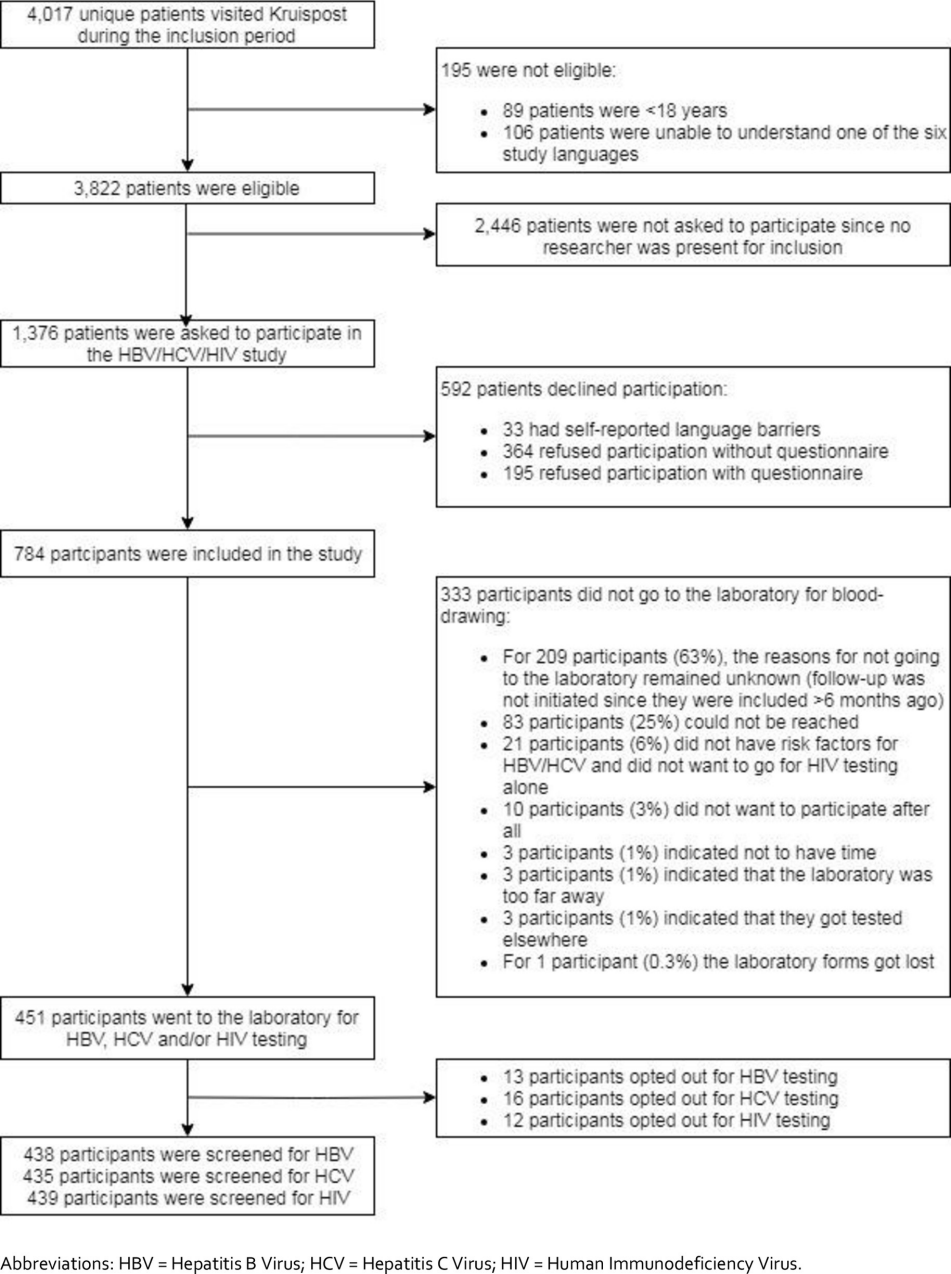
Flowchart depicting the recruitment strategy of HBV, HCV and HIV screening offered to undocumented migrants and uninsured legal residents attending Kruispost in Amsterdam, the Netherlands (N = 3,822).

In total, 4,017 patients visited Kruispost during the inclusion period. Of them, 89 (2%) were aged <18 years and 106 (3%) were unable to understand one of the six study languages, and were hence excluded. In total, 3,822 (95%) eligible patients remained. Of them, 1,376 (36%) were invited to participate and 784 (57%; 784/1376) agreed to participate in the study. The reasons for declining participation (n = 592) are described in Fig 1.

The median age of participants (N = 784) was 40 (interquartile range (IQR) 31–50) and more men than women participated (58% versus 42%, respectively). Sixty participants (8%) were uninsured legal residents and 724 participants (92%) were undocumented migrants. Participants mainly originated from Africa and Asia (35% and 30%, respectively) and the majority of participants completed secondary school (42%) or higher education (36%). Most participants reported to have arrived on a (now expired) tourist visa (45%), or as a rejected asylum seeker (18%). Fourteen percent of male participants reported having sex with men. Moreover, 14% of all participants had ≥3 sexual partners in the last 6 months (n = 104). Half of participants had never been tested for HIV (49%), HBV (55%) or HCV (61%).

Of 784 participants, 451 (58%) went to the laboratory to receive testing for at least one of the three viruses (HBV, HCV and/or HIV) (Fig 1), resulting in an overall percentage participating of 33% (n = 451/1376). Table 1 summarizes the characteristics of participants by testing uptake. Participants who refrained from blood sampling originated significantly more often from North/South America and less often from Africa, were in the Netherlands for a shorter duration, older and had a significantly higher education level than participants who were tested. In addition, participants with at least 3 sexual partners in the last 6 months underwent blood sampling significantly more often. No other variables were significantly associated with testing uptake.

**Table 1:** Characteristics of undocumented migrants and uninsured legal residents (N = 784) who participated in the Kruispost study to screen for HBV, HCV and HIV, in Amsterdam, the Netherlands, October 2018- October 2019.

	Total (N = 784)		Participants who had blood draw (N = 451)		Participants who refrained from blood draw (N = 333)		P-value*
Sociodemographic variables	n	%	n	%	n	%	
**Sex**							.993
Heterosexual men	391	50	224	50	167	50	
Men having sex with men	65	8	38	8	27	8	
Female	326	42	187	42	139	42	
Other	1	0.1	1	0.2	0	0	
**Age in years, median (IQR)**	40	(31–50)	43	(33–52)	38	(29–47)	< .001
**Age, categorized**							.001
<35 years	262	33	133	30	129	39	
35–49 years	309	39	173	38	136	41	
50–64 years	187	24	125	28	62	19	
≥65 years	26	3	20	4	6	2	
**Kruispost target population**							.931
Undocumented migrants	724	92	418	93	306	92	
Uninsured legal residents	60	8	33	7	27	8	
**Region of birth** **							.001
Europe	40	5	21	5	19	6	
Asia	234	30	131	29	103	31	
Africa	276	35	184	41	92	28	
North /South America	233	30	115	26	118	36	
**Educational level**							.027
No school	36	5	27	6	9	3	
Primary school	137	18	85	19	52	16	
Secondary school	325	42	191	43	134	41	
Higher education	280	36	146	33	134	41	
**Migration history**							
**Year of leaving country of origin, median (IQR)**	2011	(2003–2016)	2010	(2002–2016)	2012	(2005–2016)	.025
**Year of leaving country of origin**							.186
<2010	346	45	212	47	134	41	
2010–2017	335	43	182	40	153	47	
≥2018	93	12	54	12	39	12	
**Year of arrival in the Netherlands, median (IQR)**	2013	(2006–2017)	2013	(2004–2017)	2014	(2007–2017)	.043
**Year of arrival in the Netherlands**							.232
<2010	287	37	177	40	110	34	
2010–2017	316	41	176	39	140	43	
≥2018	173	22	95	21	78	24	
**Way of entering the Netherlands**							.827
(Expired) tourist/working/student visa	413	53	232	52	181	55	
Rejected asylum seeker	139	18	81	18	58	18	
EU citizen	46	6	27	6	19	6	
Illegally crossing borders	121	16	76	17	45	14	
Legally/work/other visa^$^	37	5	20	5	17	5	
Other/unknown	19	3	12	3	7	2	
**Housing situation (multiple answers possible)**							
BBB (Bed Bath Bread) facility#	58	7	32	7	26	8	.706
Friends/family	411	52	238	53	173	52	.820
Illegal rent	173	22	98	22	75	23	.791
Housing provided by charity	67	9	45	10	22	7	.095
Lives on the streets	63	8	31	7	32	10	.164
Lives in other housing##	53	7	31	7	22	7	.883
**Number of housemates**							.588
No housemates	110	14	66	15	44	14	
<3	345	45	190	43	78	48	
3–6	232	30	137	31	153	29	
≥6	81	11	50	11	13	10	
**HBV/HCV Risk factors**							
**Ever admitted/treated in a foreign hospital**	276	36	157	35	119	36	.781
**Ever had surgery abroad**	261	33	151	34	110	33	.901
**Ever received a blood transfusion**							.193
No	725	93	415	92	310	93	
Yes	45	6	25	6	20	6	
Unknown	12	2	10	2	2	0.6	
**More than 3 sexual partners in the previous 6 months**	104	14	69	16	35	11	.048
**Ever (been) paid for sex**							.072
No, never	631	81	351	78	280	84	
Yes, ever paid	127	16	82	18	45	14	
Yes, ever been paid	24	3	17	4	7	2	
**Ever injected drugs**	18	2	5	1	13	4	.010
**Infectious disease (testing) history**							
**Ever tested for HBV**							.023
No	434	56	254	56	180	54	
Unknown	120	15	78	17	42	13	
Yes, test was negative	201	26	101	22	100	30	
Yes, test was positive	12	2	10	2	2	0.6	
Yes, but results of tests are unknown	15	2	7	2	8	2	
**Ever tested for HCV**							.008
No	478	61	287	64	191	58	
Unknown	173	22	102	23	71	21	
Yes, test was negative	126	16	60	13	66	20	
Yes, test was positive	4	0.5	0	0	4	1	
Yes, but results of tests are unknown	1	0.1	1	0.2	0	0	
**Ever tested for HIV**							.683
No	387	50	219	49	168	51	
Unknown	56	7	37	8	19	6	
Yes, test was negative	323	41	186	41	137	41	
Yes, test was positive	8	1	4	0.9	4	1	
Yes, but results of tests are unknown	8	1	4	0.9	4	1	
**Mother with liver disease**							.120
No	631	81	351	78	280	84	
Yes, HBV or HCV	6	0.8	4	0.9	2	0.6	
Yes, liver cancer	6	0.8	3	0.7	3	0.9	
Unknown	139	18	92	20	47	14	
**Other family member with liver disease**							.149
No	593	76	332	74	261	79	
Yes, HBV or HCV	36	5	27	6	9	3	
Yes, liver cancer	13	2	8	2	5	2	
Unknown	140	18	83	18	57	17	

* Differences in variables according to whether or not patients received blood draw were assessed using Fisher’s Exact test for categorical data. Mann-Whitney U test was used for continuous data.

** The most common countries of birth were Brazil (n = 142), Philippines (n = 90), Nigeria (n = 80), Morocco (n = 46), Surinam (n = 43), Egypt (n = 33), Ghana (n = 33), Indonesia (n = 26), Eritrea (n = 23), India (n = 17), Colombia (n = 15), Iran (n = 14), and Somalia (n = 11).

$ Include family visa and Schengen visa.

# The bed, bath, bread regulation arranges basic emergency shelter for rejected asylum seekers provided by the Dutch government.

## Includes legal rent, housing for asylum seekers, boats, employers, hotels, crisis care, winter care and outdoors.

Missing values: Region of birth n = 1; education n = 3; year of leaving country of origin n = 5; year of arrival in the Netherlands 4; method of entering the Netherlands n = 4; number of housemates 8; admitted to foreign hospital 6; surgery abroad 2; blood transfusion 2; biological male having sex with men 1; more than 3 sexual partners in previous 6 months 21; ever (been) paid for sex 2; injected drugs 2; ever tested HBV/HCV/HIV 2; liver disease mother/family 2.

Abbreviations: IQR–inter quartile range; EU–European Union; BBB–bed bath bread; HBV–Hepatitis B Virus; HCV–Hepatitis C Virus; HIV–Human Immunodeficiency Virus.

### Prevalence of HBV, HCV and HIV infection and its determinants

A total of 130 of 438 (29,7%) participants were HBV exposed (anti-HBcAb-positive), of whom 11 participants had chronic HBV infection (HBsAg-positive), resulting in a prevalence of 2.5% (n = 11/438). Four (36.4%, n = 4/11) already knew of their chronic HBV infection. Resolved HBV infection (HBsAg-negative) was found in 27% (n = 119/438) of participants. A total of 10 participants were HCV exposed of whom 3 had chronic HCV infection (0.7%, n = 3/435) and 7 resolved HCV infection (1.6%, n = 7/435). No one knew of their HCV infection prior to participation. Five participants tested HIV positive, resulting in a prevalence of 1.1% (n = 5/439). Three participants already knew of their HIV-positive status (Table 2). No co-infections were found.

**Table 2:** Infectious disease status of participants who had blood draw (N = 435–439*).

	n	%	95%CI
**Tested for HBV (N = 438)**			
HBV exposed *(anti-HBc positive)*	130	29.7	25.4–34.2
Chronic HBV infection *(HBsAg positive & anti-HBc positive)*	11	2.5	1.3–4.5
Resolved HBV infection *(HBsAg negative & anti-HBc positive)*	119	27.2	23.1–31.6
**Tested for HCV (N = 435)**			
HCV exposed *(anti-HCV positive)*	10	2.3	1.1–4.2
Chronic HCV infection *(anti-HCV positive & HCV RNA positive)*	3	0.7	0.1–2.0
Resolved HCV infection *(anti-HCV positive & HCV RNA negative)*	7	1.6	0.7–3.3
**Tested for HIV (N = 439)**			
HIV infection	5	1.1	0.0–2.6
Known HIV infection	3	0.7	0.1–2.0
Newly diagnosed HIV infection	2	0.5	0.1–1.6

Abbreviations: CI–Confidence Interval; HBV–Hepatitis B Virus; HCV–Hepatitis C Virus; HIV–Human Immunodeficiency Virus.

* Participants could refuse testing for any of the three infections. Participants was only offered testing for both HBV and HCV when risk factors were present.

The prevalence of those HBV/HCV exposed or HIV infected did not differ between undocumented migrants and legal residents.(Tables 3–5) Sex, age, region of birth, educational level, year of leaving country of origin, way of entering the Netherlands, living in housing provided by charity, living on the streets and ever having had surgery abroad significantly differed according to HBV status (Table 3). In multivariable analysis, individuals originating from Africa and older individuals were more likely to be HBV exposed (Table 4). Variables did not differ according to HCV status (Table 5). HIV infection was more frequently observed in individuals who more recently left their country, were MSM, and had more than 3 sexual partners in the past 6 months (Table 6).

**Table 3:** Characteristics of undocumented migrants and uninsured legal residents tested for HBV (N = 438) by HBV status, in Amsterdam, the Netherlands, October 2018- October 2019.

	No HBV infection (N = 308)		Resolved HBV infection (N = 119)		Chronic HBV infection (N = 11)		P-value*
Sociodemographic variables	n	%	n	%	n	%	
**Sex**							.001
Heterosexual men	132	43	76	64	9	82	
Men having sex with men	29	10	9	8	0	0	
Female	146	48	34	29	2	18	
Other	1	0.3	0	0	0	0	
**Age in years, median (IQR)**	39.5	(32–51)	46	(36–54)	42	(29–50)	.034
**Age, categorized**							.176
<35 years	102	33	24	20	4	36	
35–49 years	114	37	48	40	4	36	
50–64 years	81	26	40	34	3	27	
≥65 years	11	4	7	6	0	0	
**Kruispost target population**							.802
Undocumented migrants	286	93	110	92	10	91	
Uninsured legal residents	22	7	9	8	1	9	
**Region of birth** **							< .001
Europe	16	5	2	2	2	18	
Asia	106	34	20	17	2	18	
Africa	90	29	78	66	7	64	
North /South America	96	31	19	16	0	0	
**Educational level**							.008
No school	14	5	9	8	0	0	
Primary school	48	17	35	29	1	9	
Secondary school	141	46	43	36	3	27	
Higher education	103	34	32	27	7	64	
**Migration history**							
**Year of leaving country of origin, median (IQR)**	2011	(2003–2016)	2007	(2001–2014)	2012	(2000–2016)	.007
**Year of leaving country of origin**							.019
<2010	131	43	69	58	5	46	
2010–2017	129	42	43	36	5	46	
≥2018	46	15	7	6	1	9	
**Year of arrival in the Netherlands, median (IQR)**	2014	(2006–2017)	2010	(2003–2016)	2012	(2006–2018)	.101
**Year of arrival in the Netherlands**							.295
<2010	110	36	56	47	4	36	
2010–2017	125	41	42	35	4	36	
≥2018	71	23	21	18	3	27	
**Way of entering the Netherlands**							.046
(Expired) tourist/working/student visa	176	58	47	39	5	45	
Rejected asylum seeker	48	16	27	23	3	27	
EU citizen	18	6	7	6	1	9	
Illegally crossing borders	40	13	30	25	2	18	
Legally/work/other visa^$^	14	5	5	4	0	0	
Other/unknown	9	3	3	3	0	0	
**Housing situation (multiple answers possible)**							
BBB (Bed Bath Bread) facility#	17	6	11	9	2	18	.093
friends/family	165	54	63	53	3	27	.242
illegal rent	70	23	22	18	3	27	.526
housing provided by charity	26	8	16	13	3	27	.048
Lives on the streets	15	5	15	13	1	9	.017
Lives in other housing##	24	8	6	5	0	0	.547
**Number of housemates**							.900
No housemates	45	15	18	16	2	18	
<3	129	43	46	40	6	55	
3–5	97	32	36	31	2	18	
≥6	32	11	16	14	1	9	
**Risk factors**							
**Ever admitted/treated in a foreign hospital**	115	38	36	31	4	36	.353
**Ever had surgery abroad**	117	38	33	28	1	9	.025
**Ever received a blood transfusion**							.160
No/unknown	285	93	116	97	11	100	
Yes	22	7	3	3	0	0	
**More than 3 sexual partners in the previous 6 months**	46	16	21	18	2	18	.703
**Ever (been) paid for sex**							.150
No, never	248	81	85	71	8	73	
Yes, ever paid	48	16	29	24	2	18	
Yes, ever been paid	11	4	5	4	1	9	
**Ever injected drugs**	3	1	1	0.8	1	9	.140

* Differences in variables according to HBV status were assessed using Fisher exact test for categorical data. Kruskal Wallis test was used for continuous data.

** The most common countries of birth were Brazil (n = 67), Nigeria (n = 59), Philippines (n = 45), Surinam (n = 28), Ghana (n = 26), Morocco (n = 24), Egypt (n = 19), Eritrea (n = 14), and Indonesia (n = 14).

$ Include family visa and Schengen visa.

# The bed, bath, bread regulation arranges basic emergency shelter for rejected asylum seekers provided by the Dutch government.

## Includes legal rent, housing for asylum seekers, boats, employers, hotels, crisis care, winter care and outdoors.

Abbreviations: HBV–hepatitis B virus; IQR–interquartile range; EU–European Union; BBB–bed bath bread.

**Table 4:** Determinants of HBV exposure among undocumented migrants and uninsured legal residents that were tested for HBV (n = 130/438) in Amsterdam, the Netherlands, October 2018—October 2019 (univariable and multivariable logistic regression analyses).

	Univariable			Multivariable		
Sociodemographics							OR	95% CI	p-value	OR	95% CI	p-value
**Sex**			< .001			
Heterosexual men	Ref					
Men having sex with men	0.48	0.22–1.06				
Female	0.38	0.24–0.60				
**Age in years**			.081			.034
<35 years	Ref			Ref		
35–49 years	1.66	0.98–2.83		1.68	0.96–2.96	
50–64 years	1.93	1.11–3.38		2.46	1.34–4.50	
≥65 years	2.32	0.82–6.53		2.03	0.67–6.17	
**Kruispost target population**			.841			
Undocumented migrant	Ref					
Uninsured legal resident	1.08	0.50–2.36				
**Region of birth** *			< .001			< .001
Europe	Ref			Ref		
Asia	0.83	0.25–2.72		0.67	0.20–2.25	
Africa	3.78	1.21–11.75		3.29	1.04–10.36	
North/South America	0.79	0.24–2.63		0.63	0.19–2.14	
**Educational level**			.017			
No school	Ref					
Primary school	1.17	0.45–2.99				
Secondary school	0.51	0.21–1.25				
Higher education	0.59	0.24–1.47				
**Migration history**						
**Year of leaving country of origin**			.004			
<2010	Ref					
2010–2017	0.66	0.43–1.02				
≥2018	0.31	0.14–0.69				
**Year of arrival in the Netherlands**			.133			
<2010	Ref					
2010–2017	0.67	0.43–1.07				
≥2018	0.62	0.35–1.08				
**Way of entering the Netherlands**			.009			
Expired tourist/working/student visa	Ref					
Rejected asylum seeker	2.12	1.22–3.677				
EU citizen	1.50	0.62–3.66				
Illegally crossing borders	2.71	1.55–4.73				
Legally/work/other visa^$^	1.21	0.42–3.51				
Other/unknown	1.13	0.29–4.32				
**Housing situation (multiple answers possible)**						
Lives in BBB (Bed Bath Bread) facility#	1.90	0.90–4.04	.101			
Lives with friends/family	0.89	0.59–1.35	.592			
Lives in illegal rent	0.81	0.49–1.35	.413			
Lives in housing provided by charity	1.86	0.99–3.49	.059			
Lives on the streets	2.74	1.31–5.73	.008			
Lives in other housing##	0.57	0.23–1.44	.213			
**Number of housemates**			.841			
No housemates	Ref					
<3	0.91	0.49–1.68				
3–5	0.88	0.46–1.68				
≥6	1.20	0.54–2.63				
**Risk factors**						
**Ever admitted/treated in a foreign hospital**	0.74	0.48–1.15	.173			
**Ever had surgery abroad**	0.58	0.37–0.91	.015			
**Ever received a blood transfusion**			.030			
No/unknown	Ref					
Yes	0.31	0.09–1.04				
**More than 3 sexual partners in the previous 6 months**	1.20	0.69–2.08	.515			
**Ever (been) paid for sex**			.106			
No, never	Ref					
Yes, ever paid	1.72	1.03–2.87				
Yes, ever been paid	1.45	0.52–4.05				
**Ever injected drugs**	1.58	0.26–9.59	.624			

* The most common countries of birth were Brazil (n = 67), Nigeria (n = 59), Philippines (n = 45), Surinam (n = 28), Ghana (n = 26), Morocco (n = 24), Egypt (n = 19), Eritrea (n = 14), and Indonesia (n = 14).

$ Includes family visa and Schengen visa.

# The bed, bath, bread regulation arranges basic emergency shelter for rejected asylum seekers provided by the Dutch government.

## Includes legal rent, housing for asylum seekers, boats, employers, hotels, crisis care, winter care, campers and forest huts.

Abbreviations: Ref–reference category; HBV–hepatitis B virus; OR–odds ratio; CI–confidence interval; IQR–inter quartile range; EU–European Union; BBB–bed bath bread.

**Table 5:** Characteristics of undocumented migrants and uninsured legal residents tested for HCV (N = 435) by HCV status, in Amsterdam, the Netherlands, October 2018- October 2019.

	No HCV infection (N = 425)		Resolved HCV infection (N = 7)		Chronic HCV infection (N = 3)		P-value*
Sociodemographic variables	n	%	n	%	n	%	
**Sex**							.273
Heterosexual men	207	49	3	43	2	67	
Men having sex with men	36	8	1	14	1	33	
Female	179	42	3	43	0	100	
Other	1	0.2	0	0	0	0	
**Age in years, median (IQR)**	42	(32–52)	51	(34–59)	52	(31–61)	.294
**Age, categorized**							.053
<35 years	126	30	2	29	1	33	
35–49 years	166	39	0	0	0	0	
50–64 years	115	27	5	71	2	67	
≥65 years	18	4	0	0	0	0	
**Kruispost target population**							.538
Undocumented migrants	394	93	6	86	3	100	
Uninsured legal residents	31	7	1	14	0	0	
**Region of birth** **							.726
Europe	20	5	0	0	0	0	
Asia	125	29	3	43	1	33	
Africa	169	40	1	14	1	33	
North /South America	111	26	3	43	1	33	
**Educational level**							.581
No school	23	5	0	0	0	0	
Primary school	78	18	1	14	2	67	
Secondary school	183	43	3	43	1	33	
Higher education	139	33	3	43	0	0	
**Migration history**							
**Year of leaving country of origin, median (IQR)**	2010	(2002–2016)	1995	(1988–2015)	2008	(2003–2015)	.369
**Year of leaving country of origin**							.967
<2010	197	47	4	57	2	67	
2010–2017	172	41	3	43	1	33	
≥2018	54	13	0	0	0	0	
**Year of arrival in the Netherlands, median (IQR)**	2013	(2005–2017)	2015	(1988–2016)	2008	(2003–2015)	.617
**Year of arrival in the Netherlands**							.958
<2010	163	39	3	43	2	67	
2010–2017	166	39	3	43	1	33	
≥2018	94	22	1	14	0	0	
**Way of entering the Netherlands**							.398
(Expired) tourist/working/student visa	221	52	5	71	1	33	
Rejected asylum seeker	76	18	1	14	0	0	
EU citizen	25	6	1	14	0	0	
Illegally crossing borders	70	17	0	0	1	33	
Legally/work/other visa^$^	18	4	0	0	1	33	
Other/unknown	12	3	0	0	0	0	
**Housing situation (multiple answers possible)**							
BBB (Bed Bath Bread) facility#	29	7	0	0	0	0	.999
friends/family	223	52	4	57	3	100	.345
illegal rent	93	22	1	14	0	0	.999
housing provided by charity	45	11	0	0	0	0	.999
Lives on the streets	30	7	1	14	0	0	.526
Lives in other housing##	29	7	1	14	0	0	.514
**Number of housemates**							.528
No housemates	63	15	1	14	0	0	
<3	173	41	5	71	3	100	
3–5	132	32	1	14	0	0	
≥6	49	12	0	0	0	0	
**Risk factors**							
**Ever admitted/treated in a foreign hospital**	152	36	2	29	1	33	.999
**Ever had surgery abroad**	149	35	2	29	0	0	.746
**Ever received a blood transfusion**							.999
No/unknown	399	94	7	100	3	100	
Yes	25	6	0	0	0	0	
**More than 3 sexual partners in the previous 6 months**	67	16	1	17	0	0	.999
**Ever (been) paid for sex**							.527
No, never	330	78	7	100	2	67	
Yes, ever paid	77	18	0	0	1	33	
Yes, ever been paid	17	4	0	0	0	0	
**Ever injected drugs**	5	1	0	0	0	0	.999

* Differences in variables according to HCV status were assessed using Fisher’s Exact test for categorical data. Kruskal Wallis test was used for continuous data.

** The most common countries of birth were Brazil (n = 67), Nigeria (n = 58), Philippines (n = 46), Surinam (n = 28), Ghana (n = 25), Morocco (n = 24), Egypt (n = 19), Eritrea (n = 14), and Indonesia (n = 14).

$ Include family visa and Schengen visa.

# The bed, bath, bread regulation arranges basic emergency shelter for rejected asylum seekers provided by the Dutch government.

## Includes legal rent, housing for asylum seekers, boats, employers, hotels, crisis care, winter care and outdoors.

Abbreviations: HCV–hepatitis C virus; IQR–interquartile range; EU–European Union; BBB–bed bath bread.

**Table 6:** Characteristics of undocumented migrants and uninsured legal residents tested for HIV (N = 439) by HIV status, in Amsterdam, the Netherlands, October 2018- October 2019.

	HIV negative (N = 434)		HIV positive (N = 5)		P-value*
Sociodemographic variables	n	%	n	%	
**Sex**					.003
Heterosexual men	219	50	0	0	
Men having sex with men	34	8	3	60	
Female	180	42	2	40	
Other	1	0.2	0	0	
**Age in years, median (IQR)**	43	(33–52)	36	(33–43)	.262
**Age, categorized**					.523
<35 years	125	29	2	40	
35–49 years	166	38	3	60	
50–64 years	123	28	0	0	
≥65 years	20	5	0	0	
**Kruispost target population**					.325
Undocumented migrants	402	93	4	80	
Uninsured legal residents	32	7	1	20	
**Region of birth** **					.361
Europe	20	5	1	20	
Asia	129	30	1	20	
Africa	173	40	2	40	
North /South America	112	26	1	20	
**Educational level**					.237
No school	27	6	0	0	
Primary school	82	19	0	0	
Secondary school	185	43	1	20	
Higher education	138	32	4	80	
**Migration history**					
**Year of leaving country of origin, median (IQR)**	2010	(2002–2016)	2016	(2012–2018)	.060
**Year of leaving country of origin**					.024
<2010	207	48	0	0	
2010–2017	173	40	3	60	
≥2018	51	12	2	40	
**Year of arrival in the Netherlands, median (IQR)**	2013	(2004–2017)	2016	(2012–2018)	.188
**Year of arrival in the Netherlands**					.155
<2010	173	40	0	0	
2010–2017	171	40	3	60	
≥2018	87	20	2	40	
**Way of entering the Netherlands**					.523
(Expired) tourist/working/student visa	225	52	3	60	
Rejected asylum seeker	76	18	0	0	
EU citizen	26	6	1	20	
Illegally crossing borders	72	17	1	20	
Legally/work/other visa^$^	20	5	0	0	
Other/unknown	12	3	0	0	
**Housing situation (multiple answers possible)**					
BBB (Bed Bath Bread) facility#	31	7	0	0	.999
friends/family	231	53	3	60	.999
illegal rent	95	22	1	20	.999
housing provided by charity	41	9	1	20	.397
Lives on the streets	29	7	0	0	.999
Lives in other housing##	29	7	0	0	.999
**Number of housemates**					.999
No housemates	62	15	1	20	
<3	184	43	2	40	
3–5	133	31	2	40	
≥6	47	11	0	0	
**Risk factors**					
**Ever admitted/treated in a foreign hospital**	151	35	0	0	.168
**Ever had surgery abroad**	147	34	1	20	.667
**Ever received a blood transfusion**					.999
No/unknown	409	94	5	100	
Yes	24	6	0	0	
**More than 3 sexual partners in the previous 6 months**	65	16	3	60	.032
**Ever (been) paid for sex**					.221
No, never	337	78	4	80	
Yes, ever paid	80	18	0	0	
Yes, ever been paid	16	4	1	20	
**Ever injected drugs**	4	0.9	1	20	.056

* Differences in variables according to HIV status were assessed using Fisher’s Exact test for categorical data. Mann-Whitney U test was used for continuous data.

** The most common countries of birth were Brazil (n = 67), Nigeria (n = 57), Philippines (n = 46), Morocco (n = 33), Surinam (n = 28), Ghana (n = 24), Egypt (n = 19), Eritrea (n = 13), and Indonesia (n = 13).

$ Include family visa and Schengen visa.

# The bed, bath, bread regulation arranges basic emergency shelter for rejected asylum seekers provided by the Dutch government.

## Includes legal rent, housing for asylum seekers, boats, employers, hotels, crisis care, winter care and outdoors.

Abbreviations: HIV–human immunodeficiency virus; IQR–interquartile range; EU–European Union; BBB–bed bath bread.

### Follow-up and treatment of diagnosed patients

Of the 11 participants with chronic HBV infection, nine (36.4%) were diagnosed with HBeAg-negative chronic infection (according to clinical practice guidelines of the European Association for the Study of the Liver [12] and were asked to return for ALT testing every six months and HBsAg testing every three years, according to the Dutch guidelines [9]. One (9.1%) was diagnosed with stage F3-F4 fibrosis and had HBeAg-negative chronic hepatitis. Two (18.2%) did not have liver fibrosis measurements for the following reasons: did not come to their appointment (*n* = 1) and unable to continue care in the Netherlands because of being expulsed to another country (*n* = 1).

All three participants newly diagnosed with chronic HCV infection were referred and followed at a hepatology referral center. They had high HCV-RNA viral loads (range 86,846–155,299 IU/mL). Only one had their fibrosis levels measured, which revealed stage F2 fibrosis. Two participants initiated treatment, the third is currently awaiting treatment.

Five persons were HIV-positive. Of the three individuals who were already aware of their HIV-seropositive status, one had never used medication since diagnosis and was referred to an HIV-treatment center. The other two known HIV seropositive persons were already using medication, one of whom was in immediate need for medication refill and was hence referred to an HIV-center for further consultation and antiretroviral prescription. The two individuals with newly diagnosed HIV were both referred to an HIV-treatment center, but only one actually arrived, started treatment, and after treatment, achieved an undetectable HIV viral load. The second person was lost to follow up.

Because of their undocumented status, some participants were initially refused care at the hospital despite efforts from the GPs and research teams and were only able to receive an appointment after extensive mediation from the researchers.

### HBV vaccination uptake

A total of 80 participants were MSM (n = 57), had ever been paid for sex (n = 16), or both (n = 7). Of them, 50 (63%) went to the laboratory and were tested for HBV infection. Of these 50 participants, 15 had either resolved or chronic HBV infection and 35 were not HBV exposed. Of the 35 latter participants, 23 were able to be contacted and/or understood the invitation for vaccination. Uptake of ≥1 HBV vaccination dose at the Public Health Service of Amsterdam was 30% (n = 7/23).

## Discussion

In this cross-sectional study among undocumented migrants and uninsured legal residents who attended an NGO healthcare facility for GP consultations in Amsterdam, the Netherlands, we found that 29.7% were HBV exposed–with 2.5% having chronic HBV infection, 64% of which were newly diagnosed; 2.3% were HCV exposed–with 0.7% having chronic HCV infection, all of which newly diagnosed; and 1.1% were HIV-positive, 40% of which were newly diagnosed.

The prevalence for chronic HBV and HCV infections found in this study are respectively 7 times and 14 times higher compared to the general Dutch population [15,21], but lower compared to studies in Italy in undocumented migrants where a prevalence of 6–9% for chronic HBV (HBsAg positive) and 3.3% for chronic HCV infection were observed [18,22]. Migrants included in these two studies predominately originated from Eastern-Europe (estimated HBV prevalence ranges from <2%-7.99%) and Sub-Saharan Africa (HBV prevalence >8%), while few individuals came from regions of lower HBV/HCV endemicity, namely North/South America (estimated prevalence <2%) [23]. Moreover, previous studies in the Netherlands that offered testing to documented migrants were targeting specific countries and observed a wide prevalence for chronic HBV and HCV [15,21], which is not comparable with our findings. Most individuals with chronic HBV infection in our study originated from Africa and tend to be older [23]. Older age was also associated with HBV exposure, which likely reflects the rollout of vaccination programs in the country of origin [24]. These findings are in line with a recent study among documented migrants in Amsterdam [3].

We observed an HIV prevalence of 1.1% in our study, which is eight times higher than that of the general Dutch population (0.1%) [4,25] yet comparable to an Italian study among undocumented migrants (1%) [18]. The United Nations goals of 95% of HIV-positive individuals knowing their status, 95% of those who know their status being engaged in care, and 95% of those who are in care having suppressed HIV replication are crucial for reducing onwards transmission [26]. The fact that we observed two newly diagnosed HIV infections and one untreated HIV-positive individual highlights some of the difficulties in achieving these goals for undocumented migrants. However, the limited number of HIV-positive individuals included in this study makes it difficult to draw conclusions on the adequacy of the HIV cascade-of-care for this specific migrant population [26].

Migrants are considered a group at risk for HBV and HCV infection according to the Dutch GP guidelines on viral hepatitis [9]. The relatively high prevalence of HBV, HCV and HIV infections in our study compared to the general Dutch population underscores that undocumented migrants are no exception to these guidelines. Coupled with the fact that screening for chronic hepatitis B and C among migrant populations is cost-effective in low-endemic settings [27], proactive case finding conducted by GPs and other healthcare workers should be considered universally in (undocumented) migrants [28]. MSM are also considered a key population at risk for HBV/HCV/HIV infections [9,29]. Our study included a very small number of migrant MSM and among them, 8% were HIV-positive, 3% had chronic HCV infection and none had chronic HBV infection. The high prevalence for HIV and chronic HCV infections in this group is worrisome, but given the small sample size, it is difficult to generalize to the overall, undocumented migrant population.

From those eligible, 32% participated in our study. Although this percentage was lower than in another integrated screening program of migrants at the tuberculosis clinic in Amsterdam (54%) [15], it was higher compared to previous non-integrated HBV and HCV screening projects targeting migrants in the Netherlands (range: 7–28%) [21,30–32]. The differences in the percentage participating across studies probably highlight the increased effectiveness of proactive HBV, HCV and HIV case finding offered by integrated existing healthcare facilities as opposed to non-integrated programs.

Participants in our study were required to have their blood drawn at an outside laboratory instead of on-site and 58% of them did make their way to the laboratory to receive testing (n = 451/784). However, on-site testing, particularly rapid testing, at the public NGO healthcare facility has been shown to remove a considerable barrier to testing uptake [33]. Unfortunately, this was not possible at Kruispost due to the absence of trained staff and high workload of nurses/doctors. Opt-out testing has also been shown to be more effective in increasing HIV testing uptake at sexual transmitted infection clinics [34] and HBV and HIV screening of migrants during antenatal care [35]. It should be noted that for many participants, their only source of healthcare was from Kruispost. These participants could view that not participating or not testing could jeopardize receiving healthcare at our center and hence why we chose not to use an opt-out testing strategy for this study. There might be several barriers to testing uptake in our study population. Previous studies among migrants in Western countries have suggested that the decision to test can be influenced by inaccurate knowledge about the disease, or being unaware that treatment options are available. In addition, stigma has also been found to withhold migrants from testing [36].

Notwithstanding the high percentage of patients able to receive appropriate follow-up, there were several noteworthy barriers. As described, some participants were initially refused care due to their undocumented status. A systematic review from 2018 supports our findings that undocumented migrants face particular problems in utilizing healthcare services in Europe [37]. A qualitative study in the Netherlands conducted in undocumented migrant women was able to substantiate personal, language, and institutional barriers to access care from the GP or hospitals for all kind of problems [38]. Failure to engage individuals with infection into care has substantial consequences further down the cascade of care, which involve lack of immediate treatment (for HIV and HCV) or assessment for treatment eligibility (for HBV) and adequate virological and clinical monitoring. Continued replication in these individuals could constitute an important reservoir for onward transmission [39,40]. Taken together, there needs to be more efficient means of ensuring that individuals diagnosed with HBV, HCV or HIV infection can be linked to care. Given the difficulties encountered at participating centers, local protocols specifying how undocumented or uninsured people can access low-threshold services for care and treatment, while including resources to secure healthcare-related funding, are needed for migrants.

HBV vaccination uptake was 30% in this study, which is lower compared to previous studies conducted in the Netherlands among MSM (50–86%) and sex workers (63%), but predominately non-migrants [41,42]. Nevertheless, this study principally aimed at infection screening in migrant populations and not necessarily HBV vaccination, while other studies conducted in the context of HBV screening observed similarly low HBV vaccine uptake [43]. An additional barrier was that participants were required to be vaccinated at the Public Health Service and not at their GP. Two studies among undocumented migrants in Italy found a lower HBV vaccination uptake of 0% to 3.4% [22,44], although they did not focus specifically on MSM or sex workers. The challenge of increasing uptake of vaccination in this migrant population likely stems from language barriers, insecurities and unfamiliarity with the health care system [19,37]. Since there were low numbers of individuals susceptible to HBV infection and the risk for further HBV transmission is unclear in this population, we cannot make any recommendations for targeting these individuals for HBV vaccination.

The main strength of our study is its inclusion of a population that has not yet been examined in previous studies in the Netherlands. We managed to reach a large group of undocumented migrants and uninsured legal residents of Amsterdam with difficult access to routine healthcare. The percentage of participants who had ever been tested for HBV/HCV or HIV support this claim. Study information was available in six different languages, which allowed us to include a very wide group of patients from many different countries. Another noteworthy feature of our study was the particular attention given to ensure linkage-to-care for individuals testing positive, which remains one of the major roadblocks to elimination, particularly for viral hepatitis.

Some limitations need to be addressed. First, we were unable to invite all patients visiting Kruispost during the inclusion period to participate in the study. Since Kruispost is a charity-based organization with limited budget and space, it was not possible to have a research associate present for inclusion during all GP-consultations. Second, only participants who visited the GP were invited, hence the study population could be more frequently in need of care. Some of these individuals might have been seeking care for their HBV, HCV or HIV infection [11,45], which would have biased prevalence estimates to be higher than expected in the target population. Third, screening for HBV and HCV were based on the presence of self-reported risk factors, hence some cases of HBV or HCV could have been missed and prevalence estimates reflect those considered at risk of viral hepatitis. Fourth, 40% of patients declined participation. Especially EU citizens were unable to be included prior to June 20, 2019 because they had to pay for care and treatment. It is therefore unclear whether our results are representative for the larger population of undocumented migrants and uninsured legal residents living in Amsterdam. Fifth, individuals from Africa were more likely to have been tested than those from South-America and given that the prevalence of chronic HBV infection is high in Africa and low to moderate in South-America [23], prevalence of chronic HBV infection could have been overestimated. In addition, MSM were more likely to receive testing than heterosexuals and since MSM were more frequently diagnosed with HIV, the prevalence of HIV-positive individuals could also have been overestimated. Sixth, we only measured anti-HBs antibodies in individuals with HBsAg or anti-HBc antibody positive status and therefore the percentage of (non)-immunized participants is unknown. Furthermore, chronic HBV infection is defined as having two consecutively positive HBsAg tests within at least 6 months. In our study, we only measured HBsAg once and thus we assumed that all those patients with HBsAg-serology had chronic infection. Lastly, absolute numbers of participants infected with HCV or HIV were low, which makes it difficult to examine risk factors. In addition, backwards selection of covariates does present with certain issues [46] and might not be optimal.

## Conclusion

To the best of our knowledge, this is the first study to examine the prevalence of chronic or resolved HBV, chronic or resolved HCV and HIV infections among undocumented migrants and uninsured legal residents in the Netherlands. The prevalence of chronic HBV, chronic HCV and HIV infections was substantially higher than in the general Dutch population, and a substantial proportions of infections was newly diagnosed. This study emphasizes the importance of proactive case finding in public health and or primary care settings and that on-site testing could likely increase screening uptake. Health care professionals must also be aware that undocumented migrants or uninsured people face barriers when referred to specialized centers for follow-up care and treatment of HBV/HCV and HIV infections. Quantitative and qualitative research focusing on access to secondary care for undocumented migrants and uninsured patients is needed to map and overcome these barriers.

## Supporting information

S1 File Questionnaire 1. Questionnaire about lifetime risk factors for HBV or HCV infection. In English.
(PDF)

S2 File Questionnaire 1. Questionnaire about lifetime risk factors for HBV or HCV infection. In Dutch.
(PDF)

S3 File Questionnaire 2. Questionnaire about sociodemographic variables and migration history. In English.
(PDF)

S4 File Questionnaire 2. Questionnaire about sociodemographic variables and migration history. In Dutch.
(PDF)

## Abbreviations

ALT: Alanine aminotransferase
CI: Confidence Interval
CSN: Citizen Service Number
EEA: European Economic Area
EPD: Electronic Patient Dossier
EU: European Union
GP: General practitioner
HBV: Hepatitis B Virus
HBsAg: Hepatitis B Surface Antigen
anti-HBcAb: anti-Hepatitis B Core antibodies
anti-HBsAb: anti-Hepatitis B Surface antibodies
anti-HBeAb: anti-Hepatitis B “e” antibodies
HBeAg: Hepatitis B “e” antigen
HCV: Hepatitis C Virus
HIV: Human Immunodeficiency Virus
IQR: Inter Quartile Range
MSM: men who have sex with men
OR: Odds Ratio
TB: Tuberculosis
WMO: Medical Research Involving Human Subjects Act
