# Supplementary material for: Hepatitis B Virus (HBV), Hepatitis C Virus (HCV) and Human Immunodeficiency Virus (HIV) infections among undocumented migrants and uninsured legal residents in the Netherlands: A cross-sectional study, 2018–2019
Source: PLoS One. 2021 Oct 29;16(10):e0258932. doi: 10.1371/journal.pone.0258932 (PMC8555813; doi:10.1371/journal.pone.0258932)
Supplement: S1 File — Questionnaire about lifetime risk factors for HBV or HCV infection. In English. (PDF) [file pone.0258932.s001.pdf]

Castor studienummer |__|__|__|__|__|__|

Questionnaire, part A

1. Are you ever tested on hepatitis B, hepatitis C or hiv?

a. Hepatitis B

- o No
- o I do not know
- o Yes --> in which year was the last time?
  |__|__|__|__| (year)

  What was the result?
  - o negative (not infected)
  - o positive (infected)
  - o I do not know

b. Hepatitis C

- o No
- o I do not know
- o Yes --> in which year was the last time?
  |__|__|__|__| (year)

  What was the result?
  - o negative (not infected)
  - o positive (infected)
  - o I don't know

c. Hiv

- o No
- o I do not know
- o Yes --> In which year was the last time?
  |__|__|__|__| (year)

What was the result?

- o I do not know
- o Hiv negative (no hiv-infection)
- o Hiv positive (hiv-infection)

When positive: Are you using medicines against hiv?

- o No, because……………………………………………………………..
- o Yes, namely………………………………………… .

2. What is your country of origin? ……………………………………………………………

3. Have you ever had surgery abroad?

All kind of operations included; also dental operations, cesarean section, cosmetic operations like a facelift or liposuction, or laparoscopic operations.

- o No
- o I do not know
- o Yes --> In which country and which year?

You can write down more countries if you got more than 1 operation.

1. In …………………………………………………………….(country) in |__|__|__|__| (year)
2. In …………………………………………………………….(country) in |__|__|__|__| (year)

4. Did you ever get a blood transfusion?

- o No
- o I do not know
- o Yes --> In which country and which year? (bloodtransfusion in Holland counts also)

If you got more times a blood transfusion in the same country: fill in the year of the first blood transfusion in that country.

1. In …………………………………………………………..…. (country) in |__|__|__|__| (year)
2. In …………………………………………………………..…. (country) in |__|__|__|__| (year)

5. As far as you know: did your mother have hepatitis B, C or liver cancer?

- o No
- o Yes, hepatitis B or C
- o Yes, livercancer
- o I do not know

6. Are there more persons in your family with liver cancer or hepatitis B or hepatitis C?
More answers possible

- □ No
- □ I do not know
- □ Yes, hepatitis B or C
- □ Yes, liver cancer

7. With what gender do you have sexual contact?

- o Men
- o Women
- o Both
- o Does not apply

8. Did you have more than 3 sexual partners the last 6 months?

- o No
- o Yes

9. Have you ever paid for sex, or did anyone ever give you something for sex (money, goods or housing)?

- □ No, never
- □ Yes, I paid for sex --> Also in the last 6 months?
  - o No
  - o Yes
- □ Yes, I got money, goods or housing for sex. --> Also in the last 6 months?
  - o No
  - o Yes

10. Have you ever injected drugs to yourself?

- o No
- o Yes
