# Supplementary material for: Hepatitis B Virus (HBV), Hepatitis C Virus (HCV) and Human Immunodeficiency Virus (HIV) infections among undocumented migrants and uninsured legal residents in the Netherlands: A cross-sectional study, 2018–2019
Source: PLoS One. 2021 Oct 29;16(10):e0258932. doi: 10.1371/journal.pone.0258932 (PMC8555813; doi:10.1371/journal.pone.0258932)
Supplement: S2 File — Questionnaire about lifetime risk factors for HBV or HCV infection. In Dutch. (PDF) [file pone.0258932.s002.pdf]

Castor studienummer |__|__|__|__|__|__|

Vragenlijst Deel A (mondelinge afname)

1. Bent u wel eens getest op hepatitis B, hepatitis C of hiv?

a. Hepatitis B

- o Nee
- o Weet ik niet
- o Ja --> in welk jaar voor het laatst?

  |__|__|__|__| (jaartal)

  Wat was de uitslag?
  - o Negatief (niet geïnfecteerd)
  - o Positief (geïnfecteerd)
  - o Weet ik niet

b. Hepatitis C

- o Nee
- o Weet ik niet
- o Ja --> in welk jaar voor het laatst?

  |__|__|__|__| (jaartal)

  Wat was de uitslag?
  - o Negatief (niet geïnfecteerd)

    Positief (geïnfecteerd)
  - o Weet ik niet

c. Hiv

- o Nee
- o Weet ik niet
- o Ja --> in welk jaar voor het laatst?

  |__|__|__|__| (jaartal)

  Wat was de uitslag?
  - o Weet ik niet
  - o Hiv negatief (geen hiv-infectie)
  - o Hiv positief (wel hiv-infectie)

Indien positief: Slikt u hiervoor medicijnen (hiv-remmers)?

- o Nee, omdat…………………………………………………………..
- o Ja, nl…………………………………………

2. In welk land bent u geboren? ………………………………………………………………

3. Bent u ooit buiten Nederland geopereerd?

Het gaat om alle soorten operaties, óók operaties aan het gebit (tanden en kiezen), keizersneden, cosmetische operaties zoals een facelift of liposuctie, of kijkoperaties waarbij in de huid wordt gesneden.

- o Nee
- o Weet ik niet
- o Ja --> In welk(e) land(en) was dat en in welk jaar?

  Meerdere landen invullen als u operaties in verschillende landen heeft ondergaan.

  1. In ……………………………..........................…………..(land) in |__|__|__|__| (jaartal)
  2. In ……………………………..........................…………..(land) in |__|__|__|__| (jaartal)

4. Hebt u ooit een bloedtransfusie gekregen? *Bloedtransfusie is het toedienen via een infuus van bloed of bloedproducten.*

- o Nee
- o Weet ik niet
- o Ja --> In welk land was dat, en in welk jaar? (bloedtransfusie in Nederland telt hier ook mee) *Als u meerdere malen een bloedtransfusie in hetzelfde land heeft gekregen, vul dan het jaar in van de eerste bloedtransfusie in dat land.*

1. In ……………………………………………………….…. (land) in |__|__|__|__| (jaartal)
2. In ……………………………………………………….…. (land) in |__|__|__|__| (jaartal)

5. Voor zover u weet, had uw moeder een hepatitis B - of hepatitis C virusinfectie, of leverkanker?

- o Nee
- o Ja, hepatitis B of C
- o Ja, leverkanker
- o Weet ik niet

6. Zijn er (andere) personen in uw familie die leverkanker of hepatitis B of C hebben?
Meerdere antwoorden mogelijk

- □ Nee
- □ Weet ik niet
- □ Ja, hepatitis B of C
- □ Ja, leverkanker

7. Met welk geslacht hebt u seksueel contact?

- o Mannen
- o Vrouwen
- o Beiden
- o Niet van toepassing

8. Hebt u meer dan 3 seksuele partners gehad in het afgelopen half jaar?

- o Nee
- o Ja

9. Hebt u ooit betaald voor seks of hebben anderen u ooit betaald voor seks (met geld, goederen of onderdak)?

- □ Nee, nooit
- □ Ja, ik heb wel eens betaald voor seks --> Gebeurde dat in de afgelopen 6 maanden?
  - o Nee
  - o Ja
- □ Ja, ik heb wel eens geld, goederen of onderdak gekregen voor seks --> Gebeurde dat in de afgelopen 6 maanden?
  - o Nee
  - o Ja

10. Hebt u ooit wel eens drugs gespoten (geïnjecteerd)?

- o Nee
- o Ja
