# Supplementary material for: Hepatitis B Virus (HBV), Hepatitis C Virus (HCV) and Human Immunodeficiency Virus (HIV) infections among undocumented migrants and uninsured legal residents in the Netherlands: A cross-sectional study, 2018–2019
Source: PLoS One. 2021 Oct 29;16(10):e0258932. doi: 10.1371/journal.pone.0258932 (PMC8555813; doi:10.1371/journal.pone.0258932)
Supplement: S3 File — Questionnaire about sociodemographic variables and migration history. In English. (PDF) [file pone.0258932.s003.pdf]

Castor studienummer |__|__|__|__|__|__|

Questionnaire B

1. What is your gender?
   - o Man
   - o Woman
   - o Other

2. What is your age?
   …………………..

3. What is your highest educational level?
   - ❏ No school
   - ❏ Primary school
   - ❏ Secondary school
   - ❏ Higher education

4. In what kind of house do you live? (More answers possible)
   - ❏ BBB (Bed, Bad, Brood)
   - ❏ Friends/family
   - ❏ Own house (illegal rent)
   - ❏ Housing provided by a charity organization such as Leger des Heils
   - ❏ I live on the street
   - ❏ Other,………………………………..

5. When did you leave your country of origin?
   Year: |__|__|__|__|

6. Since when do you live in the Netherlands?
   Year: |__|__|__|__|

7. How did you arrive in the Netherlands?
   - ❏ With a tourist visa
   - ❏ With a working visa
   - ❏ Student visa
   - ❏ As an asylum seeker (request was rejected)
   - ❏ Other, ……………………………………

8. Do you currently live with other people?
   - ❏ Yes, with ….. persons (number)
   - ❏ No

   If Yes: With who do you currently live? (please tick all boxes that are applicable)
   - ❏ I live with my partner
   - ❏ I live with (a) child(ren) below the age of 4 years
   - ❏ I live with (a) child(ren) between 4 and 17 years of age
   - ❏ I live with (a) child(ren) older than 18 years
   - ❏ I live with my parents (in law)
   - ❏ I live with other people: ………………………………………………………

9. Have you been abroad (outside of the Netherlands) for more than 24 hours in the previous 6 months?
   - ❏ Yes, namely in the following countries:
     1)……………………………………………………………………………..
     2)……………………………………………………………………………..
     3)……………………………………………………………………………..
   - ❏ No

10. Have you ever been admitted and/or treated in a foreign hospital (outside of the Netherlands)?
    - ❑ Yes, namely in the following countries during the following years:
      1) In……………………………………………(country) In ………. (year)
      2) In……………………………………………(country) In ………. (year)
      3) In……………………………………………(country) In ………. (year)
    - ❑ No

11. Are you currently using any kind of antibiotics?
    - ❑ No, no current use, but use less than 3 months ago
    - ❑ No, no current use, but use between 3 and 6 months ago
    - ❑ I don't remember when I have last used antibiotics (you can stop)
    - ❑ Never used (you can stop)
    - ❑ Yes, i am using antibiotics now

12. If you have used antibiotics in the previous 6 months, please state how many times antibiotics were prescribed in the previous 6 months:
    - ❑ 1 time
    - ❑ 2 times
    - ❑ 3 times
    - ❑ 4 times
    - ❑ 5 times or more
