# Supplementary material for: Hepatitis B Virus (HBV), Hepatitis C Virus (HCV) and Human Immunodeficiency Virus (HIV) infections among undocumented migrants and uninsured legal residents in the Netherlands: A cross-sectional study, 2018–2019
Source: PLoS One. 2021 Oct 29;16(10):e0258932. doi: 10.1371/journal.pone.0258932 (PMC8555813; doi:10.1371/journal.pone.0258932)
Supplement: S4 File — Questionnaire about sociodemographic variables and migration history. In Dutch. (PDF) [file pone.0258932.s004.pdf]

Castor studienummer |__|__|__|__|__|__|

Vragenlijst B

1. Wat is uw geslacht?
   - o Man
   - o Vrouw
   - o Anders

2. Wat is uw leeftijd?
   …………………..

3. Wat is de hoogste opleiding die u heeft gedaan?
   - o geen school
   - o basisschool
   - o voortgezet onderwijs (middelbare school)
   - o hogere schooling (zoals mbo, hbo of universiteit)

4. In welk soort huis verblijft u op dit moment? (meerdere antwoorden mogelijk)
   - o BBB (Bed, Bad, Brood)
   - o Vrienden/familie
   - o Eigen huis (illegaal gehuurd)
   - o Huis via een hulpverlenersorganisatie, zoals het Leger des Heils.
   - o Ik leef op de straat
   - o anders,………………………………..

5. Wanneer bent u vertrokken uit uw land van herkomst?
   jaar: |__|__|__|__|

6. Sinds wanneer verblijft u in Nederland?
   jaar: |__|__|__|__|

7. Hoe bent u Nederland binnengekomen?
   - o Met een toeristenvisum
   - o Met een werkvisum
   - o Met een studentenvisum
   - o Als een asielzoeker (aanvraag is afgewezen)
   - o Anders, ……………………………………………

8. Leeft u op dit moment met andere personen in een huis?
   - o Ja, met ….. personen (aantal)
   - o Nee

   Indien ja: Met wie woont u samen? (meerdere antwoorden mogelijk!)
   - □ Ik woon samen met mijn partner
   - □ Ik leef samen met mijn kinderen onder de 4 jaar
   - □ Ik leef samen met mijn kinderen tussen de 4 en 17 jaar
   - □ Ik leef samen met mijn kinderen, ouder dan 18 jaar
   - □ Ik woon samen met mijn ouders of schoonouders
   - □ Ik leef met anderen, namelijk …………………………………...

9. Bent u de afgelopen 6 maand voor meer dan 24 uur in het buitenland geweest?
   - o Ja, namelijk in de volgende landen:
     1)………………………………………………………………………..
     2)………………………………………………………………………..
     3)………………………………………………………………………..
   - o Nee

10. Bent u ooit opgenomen geweest of behandeld geweest in een buitenlands ziekenhuis?
    - Ja, namelijk in de volgende landen en de volgende jaren:
      1) In……………………………………………(land) In ………. (jaar)
      2) In……………………………………………(land) In ………. (jaar)
      3) In……………………………………………(land) In ………. (jaar)
    - Nee

11. Gebruikt u op dit moment antibiotica?
    - Nee
        - Niet op dit moment, maar wel minder dan 3 maand geleden
        - Niet op dit moment, maar wel 3-6 maand geleden
        - Ik kan me niet herinneren wanneer ik voor het laatst antibiotica heb gebruikt (u kan stoppen met invullen van de vragenlijst)
        - Nooit gebruikt (u kunt stoppen)
    - Ja

12. Als u de afgelopen 6 maand antibiotica hebt gebruikt, hoe vaak werd u antibiotica voorgeschreven de laatste 6 maand?
    - 1 keer
    - 2 keren
    - 3 keren
    - 4 keren
    - 5 keren of meer
